# Artificial Intelligence and Genomic Data Analysis: New Frontiers in Precision Medicine

**DOI:** 10.3390/ijms27156755

**Published:** 2026-07-28

**Authors:** Alexandra-Maria Blaga, Răzvan-Octavian Mihuț, Andreea-Ramona Treteanu, Octavian Andronic, Ștefan Sebastian Busnatu, Simona Dima, Viorica-Elena Rădoi

**Affiliations:** 1 Faculty of Informatics and Sciences, University of Oradea, 410087 Oradea, Romania; blagaalexandramaria@gmail.com (A.-M.B.); mihutrazvan2005@gmail.com (R.-O.M.); 2 Faculty of General Medicine, “Carol Davila” University of Medicine and Pharmacy, 050474 Bucharest, Romania; stefan.busnatu@umfcd.ro (Ș.S.B.); simona.dima@umfcd.ro (S.D.); viorica.radoi@umfcd.ro (V.-E.R.); 3 Center of Innovation and e-Health, “Carol Davila” University of Medicine and Pharmacy, 030167 Bucharest, Romania; 4 Department of Cardiology, Emergency Hospital Bagdasar-Arseni, 050474 Bucharest, Romania; 5 Center of Excellence in Translational Medicine, Fundeni Clinical Institute, 022328 Bucharest, Romania

**Keywords:** artificial intelligence, AI, genetic variants, genomic biomarkers, precision medicine, variant interpretation, cancer

## Abstract

The rapid expansion of next-generation sequencing technologies has generated unprecedented volumes of genomic data; however, translating these data into reliable and clinically actionable insights remains a major challenge in precision medicine. Artificial intelligence (AI) has emerged as a key enabling technology across the genomic medicine pipeline, supporting variant detection, variant interpretation, polygenic risk prediction, disease subtyping, biomarker discovery and treatment–response modelling. This review provides a clinically oriented, pipeline-based synthesis of contemporary AI applications in genomic medicine. Major computational paradigms, including machine learning, deep learning, ensemble methods, multimodal AI, explainable AI frameworks and emerging foundation models, are discussed in the context of their contribution to genomic analysis and clinical decision support. Particular emphasis is placed on the factors that determine model robustness and clinical utility, including dataset composition, class imbalance, label noise, calibration, ancestry representation, distributional shift and external validation. Evidence from rare genetic disorders, cardiovascular genetics and precision oncology is examined to illustrate both successful translational applications and persistent barriers to implementation. The review further analyses common sources of failure in real-world genomic AI systems, including overfitting, limited transportability across populations and sequencing environments, inadequate interpretability, and insufficient prospective validation. Ethical and regulatory challenges are discussed in relation to clinical accountability, genomic privacy, algorithmic bias and equitable implementation. Ultimately, the successful clinical translation of genomic AI will depend not only on methodological innovation, but also on rigorous validation, transparent reporting, continuous calibration, robust governance and sustained expert oversight.

## 1. Introduction

Over the past decade, precision medicine has shifted the center of gravity from diseases to individuals, with genomics becoming the backbone of this transformation. Next-generation sequencing (NGS) now enables the rapid generation of vast genomic datasets, yet the true challenge lies not in data acquisition but in converting these data into reliable clinical insight. Artificial Intelligence (AI) has moved naturally into this space: models capable of detecting subtle patterns, identifying biomarkers and predicting therapeutic responses promise to shorten the path from molecular discovery to bedside decision-making [1,2]. The stakes are concrete: earlier diagnoses, more precisely targeted therapies, and, where genetic risk is quantifiable, prevention of hereditary disease before clinical onset [2,3].

Despite this promise, genomic medicine remains complex. Most variants are benign, yet a meaningful minority exert effects too subtle to be captured by simple rules; variants of uncertain significance (VUSs) therefore remain a dominant interpretative challenge [2]. Data imbalances, including small cohorts for rare diseases and uneven population representation, limit model training and generalizability, while inconsistencies between databases such as ClinVar and Human Gene Mutation Database (HGMD) propagate label noise [3,4]. Platform heterogeneity and technical artefacts further degrade performance as models transition from clean benchmark datasets to real-world clinical environments, where variability is the norm rather than the exception [2,4].

AI now permeates every layer of the genomic workflow. Deep learning (DL) and ensemble methods have improved variant detection in challenging genomic regions, providing a more stable foundation for downstream interpretation [2]. In variant prioritization, metapredictors and modern classifiers reduce the candidate search space and focus human review where it matters most [2]. Multimodal integration has become a defining differentiator, combining genomic, transcriptomic, clinical and imaging information, illustrated by radiogenomic models that infer glioblastoma subtypes from Magnetic Resonance Imaging (MRI) and map them to transcriptomic signatures, demonstrating the power of cross-layer modelling [5,6]. In rare diseases, gene-specific models for Neurofibromin 1 (NF1) and Wilson disease show how AI can reduce the uncertainty surrounding VUSs and accelerate diagnostic resolution [7,8]. In parallel, AI-enabled refinement of polygenic risk scores (PRSs), from feature selection to nonlinear modelling, reveals genuine potential, although broad clinical utility will require further robust validation [3,9,10]. Across these developments, explainability has gained prominence, given that clinical trust cannot be sustained without transparent reasoning [2].

Yet important gaps persist. Standardized evaluation frameworks and large-scale external validation remain limited, especially in settings reflecting true clinical heterogeneity [3]. Ethical and legal considerations, from informed consent to privacy and equity, underline the need for privacy-by-design architectures and auditable model governance [11]. Data fragmentation continues to impede generalizability, making diversity in datasets, federated learning and unified reporting frameworks essential to avoid isolated pockets of performance that fail to transfer [11,12]. Finally, moving from proof-of-concept systems to clinically robust implementations requires end-to-end traceability, rigorous quality control and clearly defined processes for model updating and maintenance [2,3].

Against this backdrop, the present review adopts a pipeline-oriented and clinically translational perspective on genomic AI. We first outline the conceptual framework underlying AI in genomic medicine, including major modelling paradigms, explainability approaches, multimodal architectures and federated learning infrastructures. We then examine how AI operates across the genomic workflow, from upstream variant detection to downstream clinical decision support, with emphasis on variant interpretation, polygenic risk prediction, disease subtyping and treatment–response modelling.

Rather than focusing solely on reported performance metrics, we critically analyze why model performance differs across studies, including the effects of dataset composition, label noise, population structure, calibration and external validation. We further discuss recurrent failure modes that limit real-world implementation, together with ethical, regulatory and governance challenges associated with genomic AI deployment.

Finally, we propose practical guidance for clinicians regarding interpretation and responsible use of AI-generated genomic predictions and discuss future directions including multimodal AI, foundation models, agent-based orchestration systems and federated learning frameworks. Throughout the manuscript, emphasis is placed not only on methodological innovation, but also on the requirements necessary for safe, interpretable and clinically deployable genomic AI.

## 2. Conceptual Framework for AI in Genomic Medicine

AI in genomic medicine encompasses a heterogeneous ecosystem of computational models and infrastructures designed to transform high-dimensional molecular and clinical data into clinically actionable insights. Across precision oncology, rare genetic disorders and translational biomedical research, AI supports the integration of genomic, transcriptomic, imaging and clinical information, enabling improved disease stratification, biomarker discovery, prognostic estimation and therapeutic decision-making. However, the clinical utility of these approaches depends not only on predictive performance, but also on interpretability, robustness, fairness and secure data governance. Consequently, modern genomic AI should be viewed not as a single algorithmic paradigm, but as a layered conceptual framework integrating multiple complementary modelling strategies and computational infrastructures.

### 2.1. AI Model Landscape: From Classical Machine Learning to Foundation Models

The current landscape of AI in genomic medicine spans classical ML, DL, ensemble approaches and, more recently, foundation models trained on large-scale biomedical datasets. These paradigms differ substantially in architecture, computational requirements, interpretability and suitability for specific genomic tasks.

Classical ML algorithms, including random forests (RFs), support vector machines (SVMs), logistic regression and gradient boosting methods, remain widely used for genomic biomarker discovery, pathogenicity prediction, survival modelling and polygenic risk estimation. Their continued relevance derives from several practical advantages: they perform efficiently on structured tabular data, tolerate relatively small sample sizes, require lower computational resources and frequently provide more interpretable feature importance measures than complex neural architectures [13,14,15,16]. In genomic medicine, these methods are commonly applied to transcriptomic signatures, variant prioritization and multimodal risk stratification pipelines.

By contrast, DL architectures enable modelling of highly non-linear biological relationships that are difficult to capture using conventional ML. Convolutional neural networks (CNNs), graph neural networks (GNNs) and transformer-based architectures can integrate heterogeneous modalities including genomic sequences, transcriptomics, digital pathology and radiomics. In oncology, DL models have demonstrated particular utility for identifying latent molecular subtypes, predicting treatment response and reconstructing complex regulatory networks from multi-omic data. GNNs additionally allow biologically meaningful representation of protein interaction networks and pathway-level relationships, while transformer architectures facilitate long-range dependency modelling within genomic sequences and multimodal biomedical datasets [14,15,17].

Ensemble methods constitute an important intermediate paradigm and are frequently among the highest-performing approaches in clinical genomics. Tree-based ensembles such as Extreme Gradient Boosting (XGBoost), Light Gradient Boosting Machine (LightGBM) and stacked ensemble frameworks combine predictions from multiple weak learners to improve robustness, calibration and generalizability. In prognostic modelling and treatment–response prediction, ensembles often outperform single algorithms by reducing variance and improving stability across heterogeneous cohorts. Their compatibility with explainability frameworks has also contributed to their widespread adoption in clinically oriented genomic pipelines [15,18,19].

More recently, emerging foundation models and “generalist” medical AI systems have begun to reshape the computational landscape of precision medicine. These large pretrained architectures are trained on multimodal biomedical corpora including genomic sequences, pathology images, radiological data and clinical text, then adapted to downstream tasks through fine-tuning. Proposed applications include multi-omic integration, drug-response prediction, variant interpretation and single-cell analysis [17,20]. Although still at an early stage of clinical implementation, foundation models may enable more transferable and scalable representations of disease biology across institutions and disease domains.

### 2.2. Explainable AI in Genomic Medicine

Despite substantial advances in predictive performance, one of the principal barriers to clinical implementation of AI in genomics remains the limited interpretability of many high-performing models. In this context, explainable artificial intelligence (XAI) has emerged as a critical component of clinically acceptable genomic AI systems, particularly given increasing regulatory and ethical demands for transparency and auditability.

Among currently available XAI techniques, SHapley Additive exPlanations (SHAP) and Local Interpretable Model-agnostic Explanations (LIME) are the most widely used in genomic medicine. SHAP provides a game-theoretic framework for estimating the contributions of individual variables to model predictions, enabling ranking of genes, variants and clinical features according to their influence on a specific outcome. In cancer genomics and rare disease prediction, SHAP has been used to identify prognostic biomarkers, prioritize pathogenic variants and characterize molecular determinants of treatment resistance [15,18,19,21].

LIME complements this approach by generating local, patient-specific explanations. Rather than estimating global feature importance, LIME approximates model behaviour around an individual prediction, thereby identifying the genomic or clinical variables that most strongly influenced classification for a specific patient [18,19,21,22]. Such local interpretability may be particularly valuable in clinical decision-support settings, where clinicians require transparent justification for personalized therapeutic recommendations.

Nevertheless, current XAI approaches remain imperfect. Most methods simplify highly complex biological interactions into additive or locally approximated contributions, potentially overlooking pathway-level dependencies and emergent nonlinear behaviour characteristics of genomic systems [23]. Consequently, there is growing recognition that future XAI frameworks must become increasingly biology-aware, integrating pathway structure, regulatory networks and mechanistic constraints rather than relying exclusively on post hoc statistical attribution.

### 2.3. Multimodal AI and the Transition Toward Integrated Disease Modelling

One of the most important conceptual shifts in genomic medicine is the transition from single-omics analysis toward multimodal AI frameworks capable of integrating multiple layers of biological and clinical information. Rather than treating genomic alterations in isolation, multimodal systems combine genomics with transcriptomics, proteomics, metabolomics, radiomics, digital pathology and electronic health records to generate more comprehensive representations of disease states [15,24].

In precision oncology, multimodal DL and transformer-based architectures have demonstrated improved performance for tumor classification, prognostic estimation and prediction of therapeutic resistance compared with unimodal approaches. By integrating radiomic features with transcriptomic and genomic profiles, these models can infer molecular phenotypes non-invasively and identify clinically relevant patient subgroups that may not be detectable through conventional histopathological classification alone [14,25]. Similar strategies have been applied in rare hematological malignancies, where multimodal frameworks integrating genomic and clinical data generate more granular prognostic stratification than traditional scoring systems [15].

Beyond oncology, multimodal AI increasingly contributes to translational applications including biomaterials research, personalized therapeutics and AI-assisted gene-editing design. Integration of genomic and proteomic profiles with clinical phenotypes may facilitate patient-specific intervention strategies and improve **C**lustered **R**egularly **I**nterspaced **S**hort **P**alindromic **R**epeats (CRISPR) target selection by modelling functional genomic context more comprehensively [17,24].

Conceptually, multimodal AI represents a shift from reductionist biomarker associations toward system-level modelling of disease biology. However, this increased representational power also introduces additional challenges related to data harmonization, missing modalities, cohort heterogeneity and computational complexity, all of which remain major barriers to clinical scalability.

### 2.4. Federated Learning and Distributed Genomic AI

A central challenge in genomic medicine is that clinically valuable datasets are highly sensitive, fragmented across institutions and often inaccessible for large-scale centralized training because of regulatory and privacy constraints. Federated learning (FL) has therefore emerged as an important architectural strategy for collaborative genomic AI.

Unlike conventional centralized training, where all data are aggregated within a single repository, FL enables institutions to train models collaboratively while retaining raw genomic and clinical data locally. Only model parameters or gradients are exchanged and aggregated across participating centers. This distributed approach reduces privacy risks and facilitates compliance with data-protection regulations such as General Data Protection Regulation (GDPR) and Health Insurance Portability and Accountability Act (HIPAA) [26].

In genomic medicine, FL is particularly relevant because it enables access to larger and more diverse patient populations without requiring direct data sharing. Federated implementations of multimodal prognostic models in hematologic malignancies have demonstrated improved predictive performance through incorporation of geographically distributed cohorts while preserving patient confidentiality [15]. More broadly, FL has been proposed as a mechanism to reduce ancestry bias, improve representation of underrepresented populations and enhance model generalizability across heterogeneous healthcare systems [25,26].

Importantly, FL should not be viewed merely as a technical privacy solution, but rather as an infrastructural layer supporting scalable and equitable genomic AI. By enabling collaborative training of ML, DL, ensemble and potentially foundation models across multiple institutions, FL addresses one of the fundamental translational bottlenecks in precision medicine: the tension between data utility and patient confidentiality.

Overall, AI in genomic medicine can be conceptualized as a layered ecosystem in which complementary modelling paradigms, explainability frameworks, multimodal integration strategies and privacy-preserving infrastructures interact to support clinically actionable genomic analysis. Classical ML, DL, ensembles and emerging foundation models provide distinct yet complementary analytical capabilities; XAI methods such as SHAP and LIME enhance transparency and facilitate biomarker interpretation; multimodal AI integrates diverse biomedical signals into more holistic disease representations; and federated learning enables collaborative model development while preserving sensitive genomic data. Together, these components underpin the ongoing transition toward scalable, explainable and clinically deployable precision medicine systems.

## 3. Materials and Methods

This study was conducted as a narrative review with a clinically oriented, pipeline-based approach to summarize and critically appraise the current evidence regarding the application of AI across the genomic medicine workflow. A narrative design was considered more appropriate than a systematic review because the objective was not to answer a narrowly defined clinical question or perform quantitative evidence synthesis, but rather to integrate methodological, translational, and clinical developments spanning multiple AI applications, including variant detection, variant interpretation, polygenic risk prediction, biomarker discovery, disease subtyping, treatment–response prediction, and clinical decision support. This approach also enabled discussion of emerging technologies, methodological challenges, and future directions that are not readily captured by conventional systematic review methodology.

A structured literature search was performed in PubMed, Scopus and Web of Science using the following search formula: (“artificial intelligence” OR “AI” OR “machine learning” OR “ML” OR “DL” OR “deep learning” OR “neural network” OR “foundation model” OR “large language model”) AND (“genomic medicine” OR “genomics” OR “genetics” OR “next-generation sequencing” OR “NGS” OR “variant interpretation” OR “variant calling” OR “polygenic risk score” OR “biomarker” OR “radiogenomics” OR “multiomics” OR “pharmacogenomics” OR “treatment response”).

The primary search was restricted to studies published between 2019 and 2026. The search strategy was adapted for each database according to its indexing system and search syntax. To maximize literature coverage, backward snowball searching was performed by screening the reference lists and citation networks of eligible studies. This process also identified a limited number of landmark publications published before 2019, which were included when they provided essential methodological background, established conceptual frameworks, or represented seminal contributions to the field. Studies were selected according to their relevance to the objectives of this review. Priority was given to original research articles reporting methodological or clinical validation of AI models; prospective and retrospective cohort studies; multicenter studies; externally validated models; and implementation studies evaluating clinical utility.

High-quality reviews and meta-analyses were additionally included when they synthesized mature evidence or provided comprehensive overviews of rapidly evolving areas, thereby facilitating interpretation of the primary literature and contextualization of current evidence.

Conference abstracts, editorials, letters, opinion papers, preprints, dissertations, book chapters, and other non-peer-reviewed publications were excluded. Studies lacking sufficient methodological detail or clear clinical relevance were also excluded whenever possible.

Retrieved studies were evaluated qualitatively according to several methodological characteristics, including study design; disease area; cohort size; type of genomic or multimodal data; AI methodology; validation strategy (internal versus external); reported clinical endpoints; evidence of clinical implementation.

Rather than performing a formal risk-of-bias assessment or quantitative meta-analysis, evidence was synthesized narratively by comparing methodological approaches, identifying areas of agreement and disagreement across studies, and distinguishing analytical performance from demonstrated clinical validity and clinical utility.

As a narrative review, this work has inherent methodological limitations. Unlike systematic reviews, study selection and evidence synthesis were not conducted using predefined protocol registration, duplicate independent screening, or formal risk-of-bias assessment. Consequently, some degree of selection bias cannot be excluded. Nevertheless, the structured search strategy, use of multiple bibliographic databases, clearly defined eligibility criteria, inclusion of high-quality reviews and meta-analyses, and supplementary snowball search were intended to maximize literature coverage while providing a balanced and clinically oriented synthesis of this rapidly evolving field.

## 4. The Genomic AI Pipeline: From Sequencing Data to Clinical Decision Support

**Figure 1:**
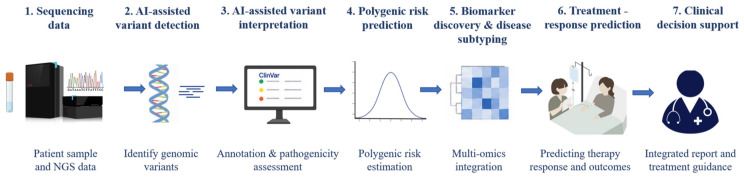
Overview of the genomic AI pipeline from sequencing data to clinical decision support. Image created using DBCLS BioIcons (https://togotv.dbcls.jp/en/pics.html, accessed on 21 June 2026), licensed under the Creative Commons Attribution 4.0 International (CC BY 4.0) license (https://creativecommons.org/licenses/by/4.0/, accessed on 21 June 2026).

The application of AI in genomic medicine is best understood not as a collection of isolated algorithms, but as a continuous analytical pipeline extending from raw sequencing data to clinically actionable decision support. AI is now embedded across multiple interconnected stages of genomic analysis, including variant detection, pathogenicity interpretation, polygenic risk prediction, molecular subtyping, biomarker discovery and treatment–response modelling (Figure 1) [27,28,29,30]. Errors, biases or calibration failures introduced at one stage may propagate downstream, amplifying uncertainty and influencing subsequent interpretation and clinical decision-making. Consequently, genomic AI should be evaluated from an end-to-end perspective that considers not only the performance of individual models, but also the interactions between upstream analytical processes and downstream clinical interpretation [2,3,11].

At the final stage of this pipeline, outputs from upstream analyses are integrated into clinical decision-support systems that generate actionable information for diagnosis, prognosis and therapeutic management. At this level, explainability, calibration, uncertainty estimation and interoperability with clinical workflows become as important as predictive accuracy. The clinical utility of genomic AI therefore depends not only on algorithmic performance, but also on transparent reporting, regulatory compliance, multidisciplinary validation and integration into existing healthcare infrastructures [31,32]. The following subsections critically examine the major components of this pipeline, with emphasis on their translational utility, methodological limitations, sources of bias and requirements for clinically reliable implementation.

Throughout this review, the term “AI-assisted” refers to computational systems that support specific stages of the genomic workflow rather than replacing expert clinical judgement. Based on the reviewed literature, AI applications in genomic medicine can be broadly grouped into three categories. The first comprises predictive machine learning and deep learning models embedded within genomic analytical pipelines, performing tasks such as variant detection, pathogenicity prediction, polygenic risk estimation, biomarker discovery, and treatment–response modelling. The second includes multimodal AI systems that integrate heterogeneous data sources, such as genomic, transcriptomic, imaging, and clinical information, to improve disease stratification and clinical decision support. The third encompasses emerging generative and agentic AI systems, including foundation models, large language models, and AI agents, which are primarily being explored for evidence synthesis, literature retrieval, workflow orchestration, and semi-automated clinical reporting rather than direct genomic analysis. Although these categories frequently overlap, they provide a useful conceptual framework for understanding the diverse roles of AI across the genomic medicine pipeline.

### 4.1. Upstream: AI-Assisted Variant Detection

Accurate variant detection represents the analytical foundation of genomic medicine, as all downstream processes, including variant interpretation, polygenic risk prediction and therapeutic decision-making, depend on the reliability of the initial variant call set. Over the past decade, AI, particularly DL-based approaches, has substantially transformed this stage of the genomic workflow by replacing conventional heuristic filtering with data-driven models capable of learning complex sequencing-error patterns directly from aligned sequencing reads. Among these, DeepVariant and related architectures, including Clair3, DeepTrio, HELLO and DNAscope, have consistently demonstrated improved analytical performance compared with traditional variant callers, particularly for germline single-nucleotide variants (SNVs) and small insertions/deletions (indels) [33,34]. For example, DeepVariant achieved 99.1% SNV accuracy on Genome in Bottle benchmark datasets, whereas the latest optimized implementation of Clair3 maintained state-of-the-art performance with SNP F1-scores of 99.32% and 99.70% on 30× Oxford Nanopore and PacBio GIAB HG003 datasets, respectively [34,35].

Current evidence indicates that the principal advantage of AI-assisted variant callers is not simply higher benchmark accuracy, but improved discrimination between true genetic variants and sequencing artefacts. Compared with conventional pipelines such as Genome Analysis Toolkit (GATK), DL-based methods consistently reduce false-positive variant calls, improve Mendelian consistency in trio sequencing, and decrease the burden of manual review without compromising sensitivity for clinically relevant pathogenic variants [36]. These improvements are particularly valuable in accredited diagnostic laboratories, where analytical precision directly influences downstream variant interpretation and diagnostic confidence.

The clinical relevance of these analytical gains is increasingly supported by studies demonstrating improved diagnostic performance in real-world sequencing applications. In germline testing, AI-assisted variant callers have identified additional pathogenic variants missed by conventional workflows while reducing false-positive findings requiring orthogonal confirmation [37]. The benefits extend beyond short-read sequencing. Integration of AI with long-read technologies, including PacBio HiFi and Oxford Nanopore sequencing, has expanded variant detection into genomic regions that have traditionally remained difficult to analyze, such as segmental duplications, homologous loci, repeat expansions and structural variants [38,39,40]. These advances suggest that AI may facilitate more comprehensive genomic analyses and reduce reliance on multiple complementary assays in selected clinical settings.

Nevertheless, the current evidence also highlights important methodological limitations that temper claims of widespread clinical readiness. Model performance remains highly dependent on sequencing platform, genomic context and variant class. While AI-assisted callers consistently achieve excellent performance for SNVs and many small indels, detection of larger indels, complex structural variants and variants located within repetitive or low-complexity genomic regions remains considerably more challenging [41,42]. Furthermore, models trained on one sequencing platform or laboratory workflow may not generalize reliably to different sequencing chemistries or clinical environments, reflecting the persistent problem of distributional shift [36,43,44]. Benchmark datasets, such as Genome in a Bottle reference materials, remain indispensable for method development but incompletely represent the diversity of clinically encountered variants, particularly rare variants and individuals from underrepresented ancestral populations [42,43,45]. Consequently, reported analytical performance may overestimate performance under routine clinical conditions.

From a translational perspective, AI-assisted variant detection should therefore be regarded as an analytical enhancement rather than a fully autonomous diagnostic solution. Current evidence supports the incorporation of DL-based variant callers into validated clinical pipelines for germline SNV and small-indel detection, where they improve analytical reliability and reduce manual review [34,36,41]. However, analytical validity should not be equated with clinical utility. Demonstrating meaningful clinical benefit requires evidence that improved variant detection translates into higher diagnostic yield, more efficient clinical workflows, or changes in patient management across diverse healthcare settings [37,38,46]. Until such prospective multicenter evidence becomes more widely available, implementation should continue to rely on platform-specific validation, orthogonal confirmation of challenging variants and integration with robust downstream interpretation frameworks.

### 4.2. AI-Assisted Variant Interpretation

Following variant detection, the principal challenge in genomic medicine is determining whether an identified variant has clinical significance. Although the majority of genetic variants are benign, distinguishing pathogenic alterations from VUSs remains one of the greatest obstacles to the clinical interpretation of genomic data. AI has substantially transformed this process by supporting pathogenicity prediction, automated evidence integration and prioritization of variants requiring expert review, thereby improving the efficiency and consistency of genomic interpretation.

Rather than replacing established interpretation frameworks, AI increasingly complements the American College of Medical Genetics and Genomics/ Association for Molecular Pathology (ACMG/AMP) guidelines by integrating diverse sources of evidence, including sequence conservation, population frequencies, protein structure, functional annotations, evolutionary constraints and, more recently, phenotype-associated information [47,48]. This transition from isolated in silico prediction tools toward quantitative evidence-based models has enabled a more standardized assessment of variant pathogenicity while reducing the subjectivity inherent to manual interpretation.

The greatest clinical impact has been observed in the interpretation of missense variants and VUSs. ML and DL models consistently improve prioritization of ambiguous variants, allowing clinical laboratories to focus confirmatory analyses on the most relevant candidates [49]. Importantly, AI has contributed not only to improved pathogenicity prediction, but also to the acceleration of variant reinterpretation. Large hereditary testing cohorts demonstrate that computational modelling now accounts for a substantial proportion of VUS reclassification events, illustrating its growing contribution to the continuous refinement of genomic diagnoses [50]. Although the proportion of reclassified variants remains relatively modest, the clinical implications are considerable given the millions of individuals undergoing genetic testing worldwide.

Beyond pathogenicity prediction, AI is increasingly used to aggregate evidence within structured interpretation frameworks. Contemporary models integrate computational predictions with ACMG/AMP evidence categories or Bayesian scoring systems, providing transparent support for evidence weighting rather than isolated pathogenicity estimates. These approaches have improved the consistency and reproducibility of variant interpretation while supporting evidence-based clinical classification [47,48,51].

The translational value of these approaches is most evident when AI supports multidisciplinary interpretation rather than autonomous decision-making. In hereditary cancer testing, AI-assisted reinterpretation has identified additional clinically actionable pathogenic variants capable of modifying surveillance strategies and cascade testing [50]. Similarly, integration of RNA sequencing with AI-based splice prediction has improved diagnostic yield in rare diseases by refining the interpretation of candidate splicing variants, highlighting the complementary value of multi-layer genomic evidence instead of sequence-based prediction alone [49]. Disease-specific interpretation frameworks have likewise demonstrated that combining computational predictions with expert-curated molecular knowledge improves classification consistency and facilitates the reinterpretation of previously unresolved variants [52].

Despite these advances, the clinical role of AI remains primarily supportive rather than autonomous. Current evidence indicates that AI is most effective in standardizing evidence assessment, prioritizing candidate variants and accelerating reinterpretation workflows, whereas the final clinical classification continues to require integration of phenotype, family history, segregation analysis, functional evidence and disease-specific expertise. Consequently, AI should be regarded as a clinical decision-support technology that enhances both the efficiency and reproducibility of variant interpretation rather than replacing expert clinical judgement.

### 4.3. AI and Polygenic Risk Prediction

Polygenic risk scores (PRSs) have emerged as one of the most promising applications of genomic medicine for predicting susceptibility to common complex diseases. Conventional PRSs aggregate the effects of numerous common genetic variants using predominantly linear statistical models; however, their predictive performance is often constrained by incomplete representation of polygenic architecture and limited integration with non-genetic determinants of disease. AI has expanded this framework by enabling genome-wide modelling, nonlinear learning and multimodal integration of genetic information with clinical, imaging, biomarker and environmental data. Rather than replacing conventional PRSs, AI primarily enhances their ability to capture complex interactions and improve individualized risk estimation.

Current evidence suggests that the greatest methodological advances arise from integrating multiple sources of information rather than from any single machine-learning architecture. Genome-wide approaches generally outperform threshold-based PRS construction by capturing a more complete representation of polygenic architecture, while multimodal models combining genetic risk with clinical variables, imaging-derived phenotypes or molecular biomarkers consistently achieve better discrimination than genetic information alone [53,54]. Similarly, ML and DL algorithms appear to provide the greatest benefit when modelling nonlinear relationships among heterogeneous data rather than when applied exclusively to PRSs [55,56].

The strongest clinical evidence has been reported for cardiovascular disease, breast cancer and type 2 diabetes, where large population cohorts, systematic reviews and biobank-based studies demonstrate that AI-enhanced risk models improve identification of individuals at increased inherited risk. In these settings, the principal clinical contribution is improved risk stratification rather than disease diagnosis, allowing more accurate classification of individuals who may benefit from earlier screening, intensified surveillance or preventive interventions [56,57,58]. Emerging applications in neurodegenerative and psychiatric disorders are also encouraging, although current evidence is focused primarily on disease stratification and multimodal decision support rather than routine clinical implementation [59,60].

AI-enhanced PRSs derive their greatest clinical value when integrated into established risk prediction frameworks. Across multiple disease areas, combining polygenic risk with conventional clinical predictors consistently provides greater predictive performance than either component alone, supporting a complementary rather than replacement role for AI-based genomic risk models [61,62]. Although discrimination metrics generally improve, evidence demonstrating corresponding improvements in patient management or clinical outcomes remains limited. Similarly, calibration and external validation are reported less consistently than discrimination metrics, and available studies suggest that model performance frequently declines when transferred across populations with different ancestry composition or clinical characteristics [63,64].

From a clinical perspective, risk stratification currently represents the most mature application of AI-enhanced polygenic prediction. Existing evidence supports the identification of subgroups with substantially different lifetime disease risk, facilitating personalized prevention strategies and risk-adapted screening programmes [63,65]. Nevertheless, improved predictive performance should not be interpreted as evidence of clinical utility. Most published studies remain observational or retrospective, and relatively few have demonstrated that implementation of AI-enhanced PRSs alters clinical decision-making or improves patient outcomes in prospective healthcare settings. Consequently, current evidence supports their use primarily as decision-support tools within comprehensive clinical risk assessment rather than as stand-alone instruments for routine implementation.

### 4.4. Disease Subtyping and Genomic Biomarker Discovery

The molecular heterogeneity of complex diseases represents one of the principal challenges of precision medicine. Patients with similar histopathological diagnoses frequently exhibit distinct genomic alterations, transcriptional programmes, therapeutic responses and clinical outcomes that cannot be adequately captured by conventional classification systems. By integrating genomic, transcriptomic, epigenomic, proteomic, imaging and clinical data, AI has emerged as a powerful framework for identifying biologically meaningful disease subtypes and discovering biomarkers with diagnostic, prognostic and therapeutic relevance.

Rather than replacing established molecular pathology methods, AI primarily functions as an integrative analytical framework capable of extracting complex patterns from high-dimensional datasets. ML algorithms have proven particularly effective for selecting informative molecular features and deriving compact genomic signatures, whereas DL approaches have expanded these capabilities through the integration of unstructured data, including digital pathology and radiological imaging [66,67,68]. The resulting multimodal models provide a more comprehensive representation of disease biology than analyses based on individual omics layers, enabling improved molecular classification and patient stratification.

These advances are most evident in precision oncology, where AI-derived molecular classifiers have substantially refined disease classification across multiple tumor types, including adrenocortical carcinoma, medulloblastoma, acute myeloid leukemia, breast, prostate and lung cancers [69,70,71]. Large clinically annotated cohorts have consistently shown that integrating multi-omic information improves prognostic stratification beyond conventional clinicopathological staging, allowing more accurate identification of patients at increased risk of recurrence, disease progression or metastasis. In several malignancies, AI-derived molecular subtypes have also revealed biologically distinct disease entities that are not apparent using traditional histopathological approaches, thereby supporting more individualized risk assessment and therapeutic planning [69,70,71].

Beyond disease classification, AI has considerably accelerated biomarker discovery. Contemporary approaches increasingly identify coordinated molecular programmes and pathway-level alterations rather than isolated biomarkers, providing a more biologically meaningful representation of disease mechanisms. Integration of transcriptomic, proteomic and methylation profiles with imaging and clinical information has enabled the identification of compact molecular signatures with prognostic and predictive value, while radiogenomic models have demonstrated the ability to infer tumor molecular phenotypes non-invasively from medical imaging. At the same time, explainable AI approaches are improving biological interpretability by linking model predictions to known signalling pathways, regulatory networks and disease-associated molecular processes, thereby increasing confidence in the clinical relevance of AI-derived biomarkers [72,73].

From a translational perspective, the most mature application of these technologies is prognostic stratification. Across several oncological settings, AI-based molecular classification consistently improves prediction of recurrence, disease-free survival, metastasis and overall prognosis beyond conventional clinical models, supporting more individualized surveillance strategies and patient management. Evidence supporting predictive biomarkers, however, remains comparatively limited. Although AI can reliably identify patient subgroups with distinct clinical outcomes, relatively few studies have demonstrated that AI-derived molecular signatures consistently predict response to specific therapies or improve treatment selection in prospective clinical settings [70,71,74]. Consequently, the clinical impact of many proposed biomarkers remains confined to patient stratification rather than treatment guidance.

Outside oncology, AI-assisted integration of multi-omic data has also shown encouraging results in neurodegenerative disorders and other complex diseases by identifying biologically relevant subgroups and improving disease characterization [72]. Nevertheless, these applications remain largely translational, and their incorporation into routine clinical practice is still limited compared with oncology, where standardized molecular classification systems and clinically annotated genomic datasets have facilitated more rapid implementation.

### 4.5. Prediction of Treatment Response

Unlike prognostic models, which estimate disease outcome irrespective of intervention, treatment–response models aim to identify patients who are most likely to benefit from a specific therapy while minimizing unnecessary toxicity.

The most mature evidence has been generated in precision oncology, where AI has been applied to predict response to chemotherapy, targeted therapies and immune checkpoint inhibitors. Across breast, colorectal, ovarian, lung and melanoma cohorts, multimodal models consistently outperform approaches based on a single data modality by combining complementary information on tumor genomics, molecular phenotype, immune microenvironment and clinical characteristics [75,76,77]. Rather than functioning as a “genome reader”, AI has evolved into an integrative framework capable of generating composite predictive biomarkers that more accurately capture treatment sensitivity than isolated molecular markers.

The greatest clinical advances have been reported for neoadjuvant chemotherapy, immunotherapy and biomarker-guided treatment stratification. In breast cancer, integration of MRI-derived radiomic features with genomic and clinicopathological variables improves prediction of pathological complete response compared with imaging alone [75]. Similarly, AI-assisted models in metastatic colorectal cancer have demonstrated the ability to predict response to chemotherapy and targeted agents while simultaneously estimating treatment-related toxicity, supporting more individualized therapeutic planning [77]. Rapid progress has also been observed in immuno-oncology, where AI increasingly models the tumor immune microenvironment to identify patients most likely to benefit from immune checkpoint inhibition beyond conventional biomarkers such as Programmed Death-Ligand 1 (PD-L1) expression [76,78].

Although oncology remains the principal area of clinical implementation, AI-assisted treatment–response prediction is expanding into other fields of genomic medicine. In rheumatoid arthritis, integration of molecular signatures with clinical characteristics has enabled identification of patients more likely to benefit from specific biologic therapies [79], while studies in schizophrenia demonstrate that combining clinical variables with genomic and epigenetic information can improve prediction of antipsychotic response [80]. These findings illustrate the broader potential of AI to support companion diagnostics and treatment stratification across diverse therapeutic settings.

The translational value of these models depends not only on predictive accuracy, but also on the clinical relevance of the endpoints being evaluated. Therapeutic response, pathological complete response, progression-free survival and treatment-related toxicity represent direct measures of treatment benefit, whereas overall survival is frequently influenced by multiple competing factors and should not be interpreted as an equivalent endpoint. Likewise, predictive biomarkers should be distinguished from prognostic biomarkers, as only the former identify differential benefit from a specific therapeutic intervention. This distinction is fundamental when assessing the real clinical value of AI-based treatment–response models.

## 5. Challenges to Clinical Translation of Genomic AI

**Figure 2:**
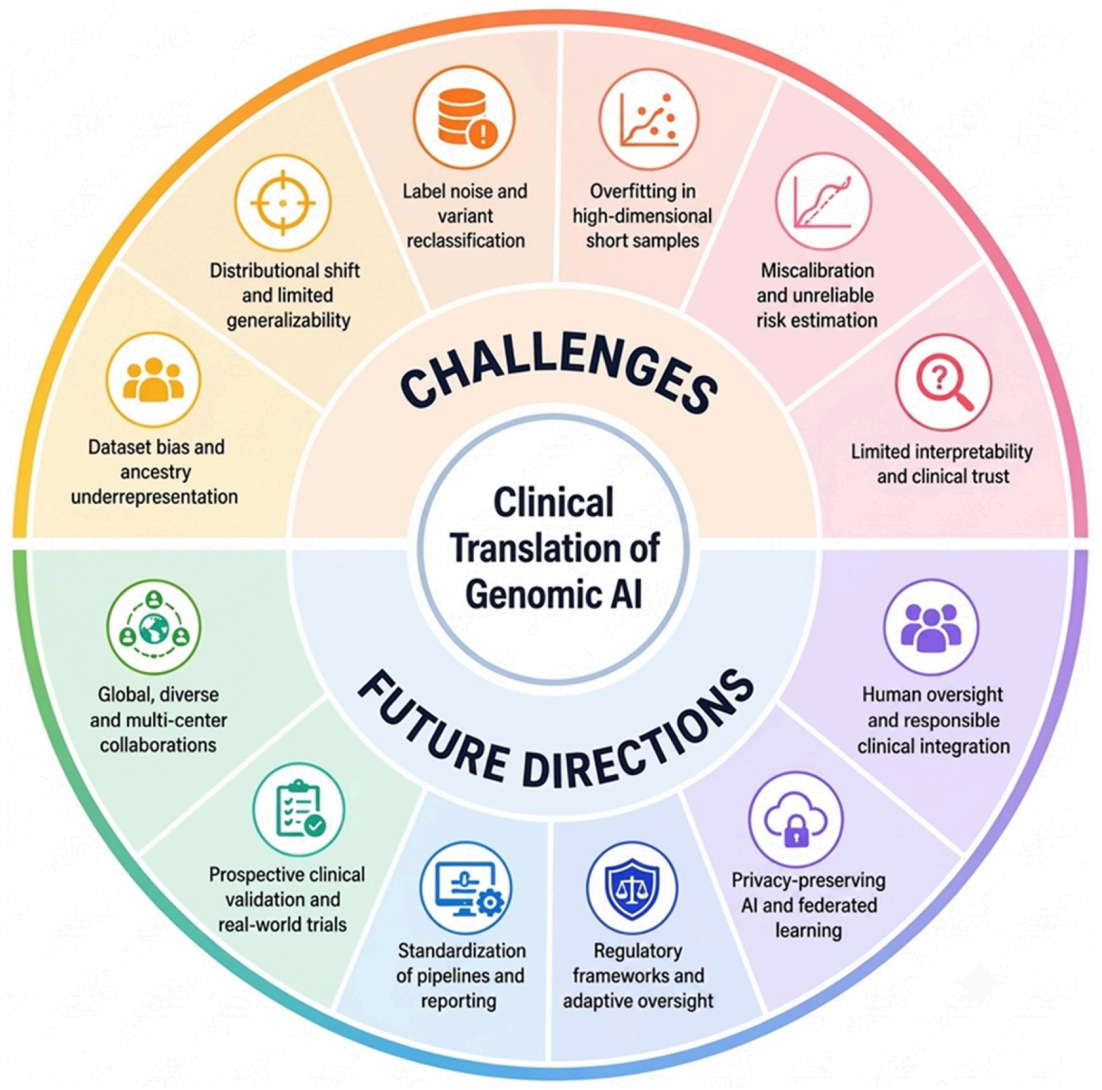
Current challenges and future directions for the clinical translation of artificial intelligence in genomic medicine.

Despite remarkable advances in predictive performance, the clinical translation of genomic AI remains constrained by several methodological limitations that compromise model robustness, reproducibility and generalizability (Figure 2). Across virtually all genomic AI applications, including variant interpretation, polygenic risk prediction, biomarker discovery, and treatment–response modelling, performance frequently declines when models are applied outside the populations or institutions in which they were originally developed.

### 5.1. Methodological Challenges Limiting Clinical Translation

Among these challenges, distributional shift represents one of the most important barriers to clinical implementation. Differences in ancestry, disease prevalence, sequencing platforms, laboratory protocols, and healthcare systems alter the statistical properties of input data, often leading to substantial reductions in predictive performance. This phenomenon has been demonstrated consistently for PRS, where predictive accuracy decreases progressively with increasing genetic distance from the population used for model development rather than simply across predefined ancestry groups. Even within broadly defined ancestry categories, considerable variability in predictive performance has been observed, highlighting that conventional ancestry labels fail to capture the genetic heterogeneity relevant for model transferability [81].

The limited portability of genomic AI models largely reflects persistent biases in genomic discovery. Most genome-wide association studies (GWAS) continue to be dominated by populations of European ancestry, resulting in training datasets that inadequately represent global genetic diversity. Consequently, AI models derived from these datasets frequently exhibit reduced performance when applied to underrepresented populations. Similar observations have been reported in South Asian, East Asian, African-descent, and highly admixed populations, where reduced predictive accuracy, altered effect sizes, and the discovery of numerous previously uncharacterized variants illustrate how incomplete population representation limits both risk prediction and variant interpretation [62,82,83]. These findings indicate that expanding ancestral diversity within genomic reference datasets is essential for improving the generalizability of AI models.

Another important limitation concerns the quality and stability of the labels used for supervised learning. Many genomic AI models rely on variant classifications derived from public databases such as ClinVar, which has become one of the principal reference resources for pathogenicity interpretation and benchmarking of AI-assisted variant classification. However, ClinVar annotations evolve continuously as new clinical evidence becomes available, and differences in curation practices, population representation and interpretation criteria may introduce label noise that propagates into model development. Beyond ClinVar, standardized benchmarking resources such as Genome in a Bottle (GIAB) provide high-confidence reference genomes for validating and comparing variant-calling algorithms, whereas population-scale resources such as the UK Biobank enable the development and external validation of AI models in large, deeply phenotyped cohorts [84,85]. Together, these resources improve reproducibility and comparability across studies, although they still face important limitations, including incomplete representation of global genetic diversity and continuously evolving genomic annotations. Differences in curation practices, population representation, and variant interpretation across databases introduce label noise that propagates directly into model development. Large-scale analyses from the All of Us Research Program further suggest that observed differences in pathogenic variant frequencies across ancestry groups often reflect unequal accumulation of genomic knowledge rather than genuine biological variation, emphasizing that inaccuracies in reference databases may contribute as much to prediction errors as the algorithms themselves [86].

Although methodological innovations such as transcriptome-informed polygenic scores, multimodal learning, and ancestry-aware modelling have partially improved model portability, their benefits remain modest and inconsistent across independent cohorts [87]. Comparative evaluations of multiple PRS construction strategies continue to demonstrate that no single modelling approach consistently overcomes the challenges imposed by population diversity or heterogeneous clinical datasets.

Collectively, current evidence indicates that the principal obstacles to clinical translation are not primarily related to algorithmic sophistication, but rather to the quality, diversity, and representativeness of the underlying data.

### 5.2. Ethical and Legal Challenges

The clinical implementation of genomic AI raises ethical and legal challenges that extend beyond algorithmic performance. Unlike many other medical data types, genomic information is inherently identifiable, biologically persistent, and shared among biologically related individuals, making issues of privacy, informed consent, data ownership, and equitable access central to the responsible deployment of AI in precision medicine. Consequently, successful clinical translation depends not only on methodological robustness, but also on governance frameworks capable of maintaining public trust while enabling responsible data sharing.

One of the greatest ethical challenges concerns the secondary use of genomic data for AI development. Because robust AI models require large, diverse, and continuously updated datasets, clinical genomic information is increasingly reused for algorithm training, external validation, and multicenter collaborations. However, multiple international studies demonstrate that patients generally support genomic data sharing only under specific conditions. Trust is consistently higher when data are used by healthcare professionals or academic institutions than by commercial organizations, and willingness to share genomic information declines when future uses, international collaborations, or commercial partnerships are insufficiently explained [88,89]. These findings suggest that public acceptance of genomic AI depends less on data sharing itself than on transparent governance, clearly defined purposes of use, and sustained institutional trust.

Current evidence also indicates that informed consent should be regarded as a dynamic rather than static process. Although most patients undergoing clinical genomic testing agree to data sharing for research purposes, many express a desire to retain control over future secondary uses of their data, particularly when information may be reused for applications not anticipated during the original consent process [88]. Furthermore, several studies report substantial variability in patient understanding of genomic data sharing, with lower comprehension observed among individuals with language barriers or limited genomic literacy [89,90]. Educational interventions alone appear insufficient to overcome these challenges, highlighting the need for continuous communication strategies that promote meaningful rather than purely administrative consent.

Privacy represents an equally important concern because complete anonymization of genomic information is fundamentally difficult. Advances in AI and large-scale data integration increase the possibility of re-identification even after conventional de-identification procedures, particularly when genomic data are combined with clinical, demographic, or publicly available information. Although technical approaches such as federated learning, differential privacy, secure multiparty computation, and encrypted data-sharing infrastructures have emerged as promising strategies for reducing privacy risks, current evidence suggests that these methods mitigate rather than eliminate the possibility of unauthorized data reconstruction or secondary misuse [91,92]. Consequently, technical safeguards must be complemented by robust legal and institutional governance mechanisms.

Ethical concerns also extend to fairness and health equity. The underrepresentation of non-European populations within genomic databases not only limits predictive performance, but may also contribute to unequal access to precision medicine. Poorly calibrated AI models can systematically underestimate or overestimate disease risk in underrepresented populations, thereby reinforcing existing disparities in screening, diagnosis, and therapeutic decision-making [83]. Increasing evidence therefore supports viewing ancestry bias not merely as a methodological limitation, but also as an ethical challenge with direct implications for distributive justice. Addressing these inequities requires greater diversity in genomic reference datasets, transparent reporting of population composition, and active engagement of historically underrepresented communities throughout the development and validation of genomic AI systems.

Ultimately, ethical implementation of genomic AI depends on maintaining a balance between innovation and patient protection. Public trust is strengthened when governance frameworks emphasize transparency, ongoing patient engagement, privacy protection, equitable representation, and clear accountability regarding the collection, sharing, and future use of genomic information. These principles should therefore be considered fundamental prerequisites for the responsible clinical translation of AI-assisted genomic medicine rather than secondary considerations addressed after technological development.

## 6. Future Directions: Toward Integrated and Clinically Deployable Genomic AI

Future progress in genomic AI will likely depend less on isolated improvements in predictive accuracy and more on the development of integrated, clinically deployable systems capable of combining heterogeneous biomedical information while remaining interpretable, robust and operationally feasible. Although recent advances in multimodal AI, foundation models and autonomous AI agents have generated substantial interest, their clinical value in genomic medicine remains largely prospective and should be interpreted cautiously.

One major direction is the continued expansion of multimodal AI. Current evidence suggests that integrating genomics with transcriptomics, epigenomics, radiomics, pathology, proteomics and electronic health record data may improve disease stratification and treatment–response prediction beyond single-omics approaches. In oncology, multimodal systems already demonstrate improved performance for molecular subtyping and immunotherapy prediction compared with isolated biomarkers such as PD-L1 or tumor mutational burden alone [93]. Future models will likely move toward longitudinal multimodal frameworks capable of incorporating dynamic clinical and molecular changes over time rather than relying on static baseline datasets.

Another rapidly evolving area involves foundation models and “generalist” biomedical AI architectures. Inspired by large language models and transformer-based systems, these approaches aim to pretrain large-scale models on genomic sequences, transcriptomic data, imaging, pathology and clinical text before adapting them to downstream tasks such as variant interpretation, biomarker discovery or drug-response prediction [4,5]. In principle, foundation models could reduce dependence on narrowly disease-specific pipelines and improve transfer learning across rare diseases or underrepresented clinical scenarios. However, substantial challenges remain, including computational cost, interpretability, dataset harmonization and the risk that large pretrained systems may amplify existing biases present in genomic repositories.

The emergence of agentic AI systems and orchestration frameworks may further transform how genomic workflows are organized. Unlike conventional predictive models that perform isolated analytical tasks, agentic AI systems are designed to autonomously coordinate multiple specialized models, iteratively reason across complex problems, and dynamically interact with external tools and knowledge sources. In genomic medicine, such systems could coordinate sequential stages of genomic analysis, including variant calling, annotation, phenotype integration, literature retrieval, ACMG evidence aggregation and therapeutic prioritization. Such orchestrated systems could potentially reduce fragmentation across current genomic pipelines and support semi-automated clinical reporting. Nevertheless, these architectures also introduce additional complexity, increasing the risk of error propagation, hidden dependencies between models and reduced transparency regarding how final recommendations are generated. Consequently, reliable orchestration will require rigorous validation, traceability and clear human oversight mechanisms before routine clinical adoption [94].

Another promising direction is the integration of causal AI and AI-assisted laboratory automation into genomic medicine. Whereas most current AI models are primarily optimized for prediction, causal AI aims to infer mechanistic relationships between genetic variants, molecular pathways and clinical outcomes, thereby complementing predictive modelling with biologically interpretable insights. Such approaches could improve model robustness, facilitate therapeutic target discovery and support more informed clinical decision-making. In parallel, AI-assisted laboratory automation has the potential to streamline genomic workflows by supporting automated sequencing quality control, sample processing, variant validation and high-throughput functional assays. The convergence of intelligent analytical models with increasingly automated laboratory platforms may accelerate the translation of genomic discoveries into clinical practice while improving reproducibility and reducing operator-dependent variability. Nevertheless, both causal AI and AI-assisted laboratory automation remain at relatively early stages of development in genomic medicine and will require extensive validation before widespread clinical adoption [94].

FL is also likely to remain an important future infrastructure for genomic AI because it addresses a central limitation of genomic medicine: the inability to freely centralize sensitive patient-level data across institutions. By enabling collaborative training while maintaining local data storage, federated learning may facilitate development of larger and more diverse genomic models while reducing some privacy concerns. However, current enthusiasm surrounding federated learning should be tempered by practical limitations. Federated systems remain vulnerable to heterogeneity in sequencing platforms, annotation standards, preprocessing pipelines and clinical labels across participating centres. Communication costs, model drift, uneven institutional resources and residual privacy vulnerabilities also remain substantial operational challenges. Importantly, federated learning does not automatically eliminate bias if participating datasets remain demographically unbalanced or inconsistently curated.

More broadly, future advances in genomic AI will likely depend on improving robustness, calibration and clinical integration rather than pursuing increasingly complex architectures alone. Many current models remain constrained by retrospective training datasets, inconsistent reporting standards and insufficient prospective validation. Consequently, the next phase of genomic AI may shift from proof-of-concept model development toward clinically supervised learning systems capable of continuous recalibration, transparent auditing and integration into multidisciplinary care pathways.

## 7. Practical Guidance for Clinicians

**Figure 3:**
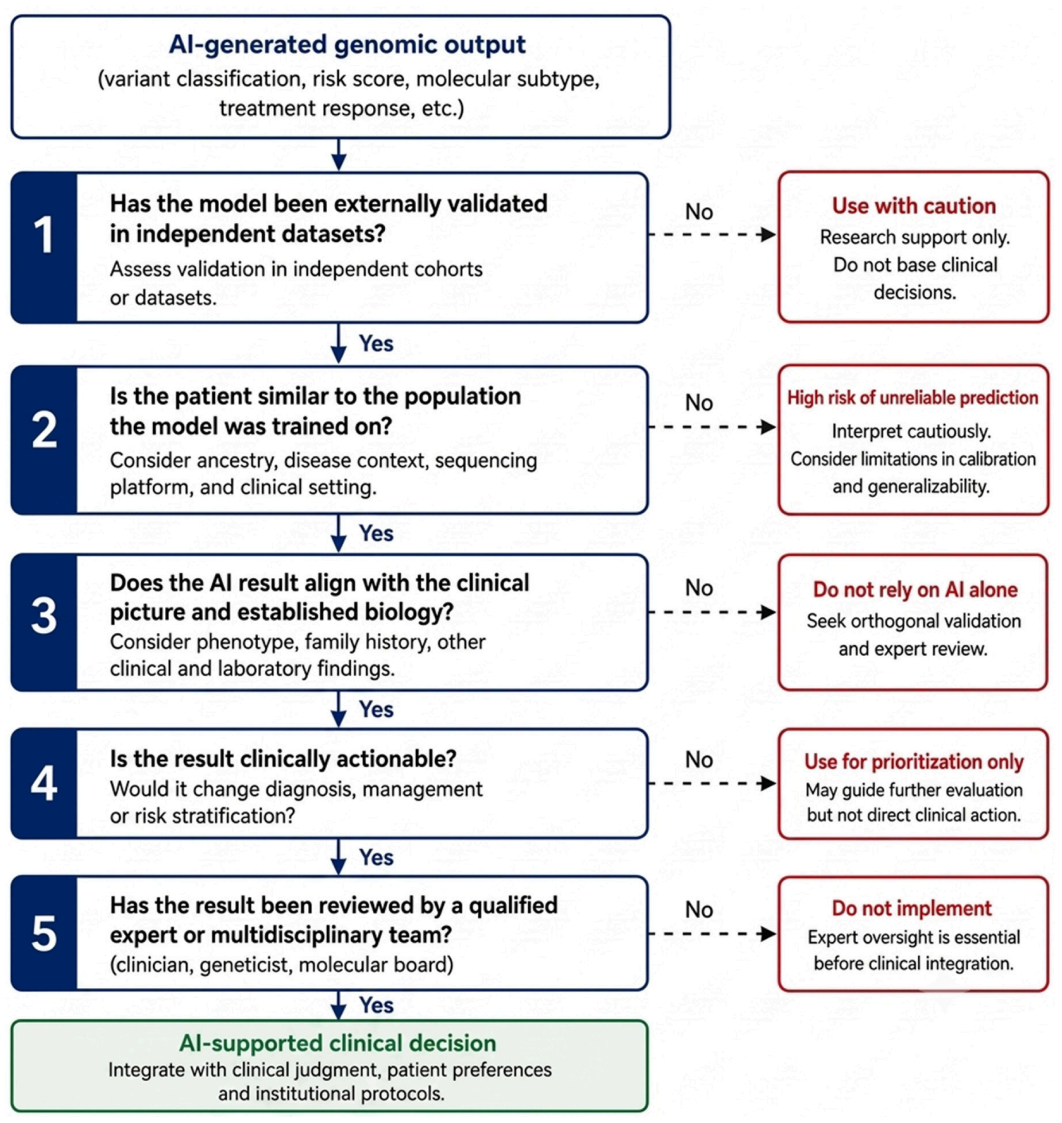
Clinical decision flow for AI-assisted genomic interpretation. Proposed pragmatic framework illustrating how clinicians may evaluate the reliability and clinical applicability of AI-generated genomic outputs before integration into patient care. The flow emphasizes key safeguards including external validation, population applicability, biological concordance, clinical actionability and expert review, highlighting situations in which AI outputs should be interpreted cautiously or used only for prioritization. The framework supports a “human-in-the-loop” approach in which AI augments, but does not replace, clinical expertise. Abbreviations: AI, artificial intelligence.

At the current stage of development, genomic AI should be viewed primarily as a clinical decision-support technology rather than an autonomous diagnostic system. The most reliable use cases are those in which AI reduces analytical burden, prioritizes complex information and assists expert interpretation, while final clinical decisions remain supervised by clinicians, molecular geneticists and multidisciplinary teams (Figure 3).

In practice, AI is currently most useful in high-dimensional and time-intensive tasks such as variant prioritization, ACMG evidence aggregation, splice prediction, molecular subtyping and multimodal risk stratification. In these contexts, AI can substantially accelerate interpretation workflows and improve consistency, particularly when analyzing large sequencing datasets or integrating multiple omics layers. Conversely, clinicians should be cautious when AI outputs require contextual reasoning that extends beyond the training data, including phenotype specificity, familial segregation, de novo interpretation, mosaicism or functional biological consequence. These domains still depend heavily on clinical expertise and orthogonal validation.

A critical practical principle is that strong retrospective performance does not automatically imply clinical reliability. Clinicians should therefore evaluate whether a model has undergone external validation, whether calibration has been reported and whether the population used for training resembles the patient population in which the tool is being applied. Models trained predominantly on highly selected cohorts or limited ancestry groups should not be assumed to generalize safely across populations.

Similarly, AI-generated probabilities should not be interpreted as definitive biological truth. Genomic AI systems estimate statistical likelihood based on learned patterns and remain vulnerable to dataset bias, label instability and distributional shift. High-confidence predictions may therefore still be incorrect, particularly for rare variants, underrepresented populations or uncommon clinical scenarios. Whenever AI outputs conflict with phenotype, family history or established disease biology, clinical judgment and orthogonal evidence should take precedence.

For polygenic risk prediction, current evidence supports the use of PRSs primarily as adjunctive risk modifiers rather than standalone clinical decision tools. PRSs may help refine risk estimation in selected contexts, particularly when integrated with conventional clinical factors, but they should not independently determine screening strategies, treatment escalation or preventive interventions in the absence of robust prospective validation.

In oncology, AI-derived treatment–response models should be interpreted cautiously, especially when developed from retrospective cohorts or when predictions may influence therapies associated with significant toxicity or irreversible consequences. At present, the most appropriate role for these systems is to support prioritization and hypothesis generation within multidisciplinary molecular tumor boards rather than autonomous treatment selection.

Clinicians should also recognize that explainability does not necessarily guarantee biological validity. Feature-attribution methods such as SHAP or LIME may improve transparency, but they provide statistical explanations rather than mechanistic proof. Explainability tools should therefore support, rather than replace, biological and clinical reasoning.

Overall, the safest approach to genomic AI is neither uncritical adoption nor complete rejection. Current evidence supports a “human-in-the-loop” model in which AI augments expert interpretation while clinicians remain responsible for contextualization, verification and final decision-making. In this framework, AI functions most effectively as a tool for prioritization, integration and workflow support rather than as an independent source of clinical authority.

## 8. Conclusions

AI is increasingly transforming genomic medicine, not as a single technology, but as an integrated computational framework supporting variant detection, pathogenicity interpretation, polygenic risk prediction, molecular subtyping, biomarker discovery, and treatment–response modelling. Current evidence indicates that AI can substantially improve prioritization, scalability, and multimodal data integration across genomic workflows, particularly in settings characterized by high-dimensional and analytically complex datasets. However, the clinical value of these systems depends less on isolated retrospective performance metrics than on robustness, calibration, interpretability, and consistent performance across heterogeneous real-world clinical environments.

Although AI has demonstrated considerable analytical advances across multiple domains of genomic medicine, translating these advances into routine clinical practice remains challenging. Persistent limitations, including distributional shift, ancestry bias, label instability, insufficient external validation, and limited prospective evidence, continue to restrict widespread clinical implementation. Consequently, current evidence supports the role of genomic AI primarily as a clinician-supervised decision-support tool rather than as an autonomous diagnostic system.

Future progress will depend on multimodal integration, multicenter collaboration, and the development of clinically deployable infrastructures capable of combining genomics with transcriptomics, proteomics, imaging, digital pathology, and longitudinal clinical data. Emerging foundation models and agent-based orchestration systems may further streamline currently fragmented genomic workflows, although important challenges related to validation, transparency, governance, and equitable implementation remain to be addressed. Ultimately, the successful integration of AI into routine genomic medicine will require not only continued methodological innovation, but also rigorous prospective evaluation, standardized reporting, representative datasets, and sustained human oversight to ensure safe, reliable, and clinically meaningful implementation.

## Data Availability

No new data were created or analyzed in this study. Data sharing is not applicable to this article.
